# Supplementary material for: Influence of the Graft Length on Nanocomposite Structure and Interfacial Dynamics
Source: Nanomaterials (Basel). 2023 Feb 16;13(4):748. doi: 10.3390/nano13040748 (PMC9960434; doi:10.3390/nano13040748)
Supplement: Supplementary file 1 [file nanomaterials-13-00748-s001.zip › nanomaterials-2199245-supplementary.pdf]

Supporting Information

Influence of the Graft Length on Nanocomposite Structure and Interfacial Dynamics

Anne-Caroline Genix,[1,*] Vera Bocharova,[2] Bobby Carroll,[3] Philippe Dieudonné-George,[1] Edouard Chauveau,[1] Alexei P. Sokolov,[2,3,4] and Julian Oberdisse [1,*]

[1]*Laboratoire Charles Coulomb (L2C), Université de Montpellier, CNRS, F-34095 Montpellier, France*

[2]*Chemical Sciences Division, Oak Ridge National Laboratory, Oak Ridge, TN 37831, USA*

[3]*Department of Physics, University of Tennessee, Knoxville, TN 37996, USA*

[4]*Department of Chemistry, University of Tennessee, Knoxville, TN 37996, USA*

* Corresponding authors: anne-caroline.genix@umontpellier.fr; julian.oberdisse@umontpellier.fr

I. Thermogravimetric analysis (TGA) data

The TGA data acquired from the surface-modified nanoparticles in suspension are presented in Figure S1. The weight loss between 473 and 873 K was used to estimate the grafting density.

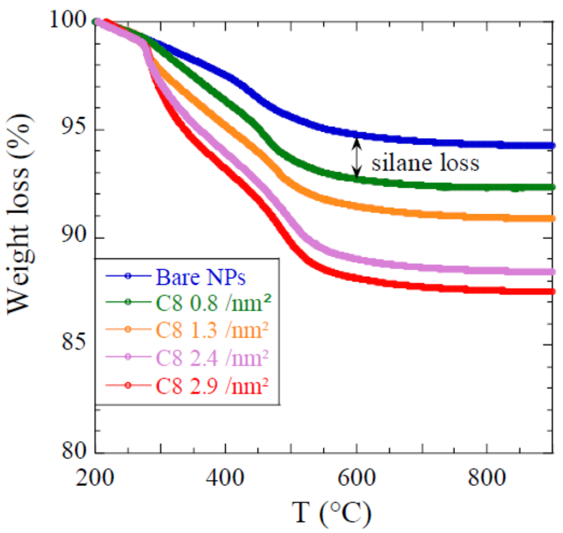

Figure S1. Percentage of weight loss versus temperature for the surface-modified NPs with different grafting densities in ethanol.

II. Small-angle X-ray scattering (SAXS) data of the colloidal suspensions

The colloidal suspension with bare silica nanoparticles (NPs) was characterized by SAXS after dilution in ethanol. The scattered intensity was fitted with a log-normal distribution of spherical objects (R_0 = 8.4 nm, σ = 18%) in Figure S2a demonstrating a good NP dispersion in ethanol.

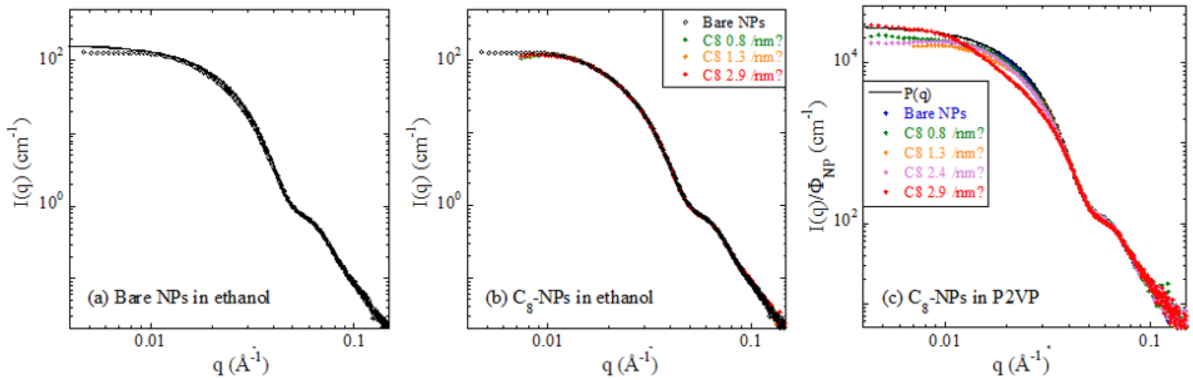

Figure S2. SAXS intensities (symbols) and modeling by log-normal distribution (line) of bare spherical particles of colloidal silica suspension (**a**) and of the C_8 surface-modified NPs (**b**) diluted in ethanol (Φ_{NP} = 0.3%v). (**c**) SAXS intensities (symbols) of P2VP nanocomposites filled with bare and surface-modified NPs at low volume fraction (Φ_{NP} = 2.0, 1.9, 1.9, 1.5, and 0.3%v, for bare and C_8-NPs with grafting density from 0.8 up to 2.9 nm^{-2}). The line is the NP form factor in P2VP/silica contrast for comparison with the PNC data.

The experimental intensities of the bare and surface-modified NPs suspended in ethanol and at low concentration in P2VP are given in Figures S2b and S2c, respectively. At intermediate q, around the form factor oscillation (ca. 0.06 Å^{-1}), the superposition of the curves in Figure S2c is less perfect than in ethanol with a slight shift of the oscillation towards higher q and a lower exponent of the high-q power law for the sample with highest grafting (2.9 /nm^2), suggesting that some modification of the scattering length density around the particles is ultimately visible in the polymer matrix. Although these deviations are quite small and not visible at low grafting density, they present a first structural evidence of the impact of grafting. Several curves are regrouped in the low-q range, while the highest grafting density has a clearly different shape around 0.02 Å^{-1} (correlation hole), indicating a change in NP interactions towards short-range repulsion.

III. Additional broadband dielectric spectroscopy (BDS) data

In Figure S3, the dielectric loss functions are shown at 423 K for various surface modifications at 15%v and 20%v of silica in nanocomposites.

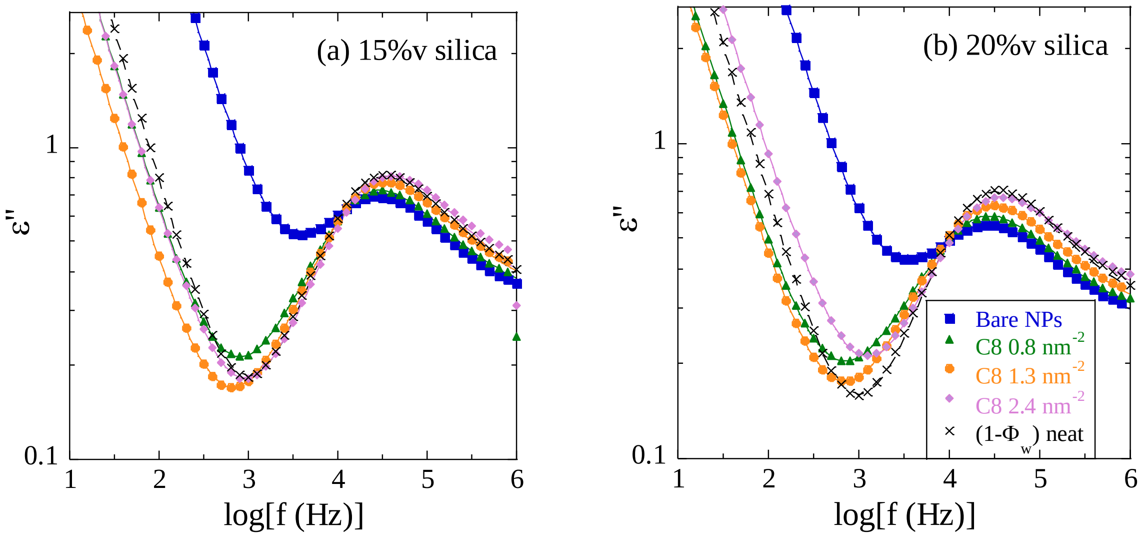

Figure S3. Dielectric loss spectra of P2VP PNCs with different surface modifications of the silica NPs for the series with Φ_{NP} = 15 (**a**) and 20%v (**b**) at 423 K. The solid lines represent the ILM fit with additional MWS and conductivity terms at low frequency and the β-process at high frequency.

The PNC BDS spectra in Figure 2 and Figure S3 are described using the interfacial layer model (ILM), the equations of which are recalled below. The fit parameters are given in Table S1.

The complex permittivity of PNC, $\varepsilon^*_{\mathrm{PNC}}$, considering that an interfacial layer is present between the filler particles and the bulk polymer is given by [19]

$$9\varepsilon^*_{\mathrm{PNC}}(\omega) = \frac{\Phi_{\mathrm{NP}}\varepsilon^*_{\mathrm{NP}}(\omega)+\Phi_{\mathrm{IL}}^{\mathrm{PNC}}\varepsilon^*_{\mathrm{IL}}(\omega)\mathrm{R}^*+\Phi_{\mathrm{b}}\varepsilon^*_{\mathrm{b}}(\omega)\mathrm{S}^*}{\Phi_{\mathrm{NP}}+\Phi_{\mathrm{IL}}^{\mathrm{PNC}}\mathrm{R}^*+\Phi_{\mathrm{b}}\mathrm{S}^*} \quad \text{(S1)}$$

where $\varepsilon^*_{\mathrm{NP}}$, $\varepsilon^*_{\mathrm{IL}}$, and $\varepsilon^*_{\mathrm{b}}$ are the complex dielectric functions of silica nanoparticles, interfacial layer, and bulk polymer (matrix), respectively. In the case of spherical particles, R* and S* are given by

$$\mathrm{R}^* = \frac{2\varepsilon^*_{\mathrm{IL}}(\omega)+\varepsilon^*_{\mathrm{NP}}(\omega)}{3\varepsilon^*_{\mathrm{IL}}(\omega)} \quad \text{(S2)}$$

$$\mathrm{S}^* = \frac{[\varepsilon^*_{\mathrm{IL}}(\omega)+2\varepsilon^*_{\mathrm{b}}(\omega)][\varepsilon^*_{\mathrm{NP}}(\omega)+2\varepsilon^*_{\mathrm{IL}}(\omega)]+2\mathrm{d}[\varepsilon^*_{\mathrm{IL}}(\omega)-\varepsilon^*_{\mathrm{b}}(\omega)][\varepsilon^*_{\mathrm{NP}}(\omega)-\varepsilon^*_{\mathrm{IL}}(\omega)]}{9\,\varepsilon^*_{\mathrm{IL}}(\omega)\,\varepsilon^*_{\mathrm{b}}(\omega)} \quad \text{(S3)}$$

with $\mathrm{d} = \frac{\Phi_{\mathrm{NP}}}{\Phi_{\mathrm{NP}}+\Phi_{\mathrm{IL}}^{\mathrm{PNC}}}$ and $\Phi_{\mathrm{NP}} + \Phi_{\mathrm{IL}}^{\mathrm{PNC}} + \Phi_{\mathrm{b}} = 1$. Φ_{NP}, $\Phi_{\mathrm{IL}}^{\mathrm{PNC}}$, and Φ_{b} are the volume fraction of silica nanoparticles, interfacial layer, and bulk polymer (matrix), respectively. The dielectric spectra were fitted using $\varepsilon^*_{\mathrm{NP}} = 3.9$ (constant) in our experimental temperature and frequency range. [17] $\varepsilon^*_{\mathrm{IL}}(\omega)$ and $\varepsilon^*_{\mathrm{b}}(\omega)$ were both described by the sum of two Havriliak-Negami functions for the α- and β-processes. For the bulk polymer function, $\varepsilon^*_{\mathrm{b}}(\omega)$, the same spectral shape parameters, amplitude and time-scale as in the neat polymer were used, i.e., this function was completely fixed. In all cases, the β-process was extrapolated from the low-T data.

Table S1. ILM fit parameters of silica-P2VP PNCs using eqs (S1-S3).

		$\mathrm{Log}[\tau_{max,IL}$ (s)]	$\tau_{max,\,IL}/\,\tau_{max,\,neat}$	$\Delta\varepsilon_{IL}$	γ_{IL}	δ_{IL}	Φ_{IL}^{PNC} (%v)
**15%v-series**	**Bare**	-4.776	3.22	1.66	0.59	1	31.0
	**C_8 0.8/nm²**	-4.922	2.30	1.67	0.68	1	29.5
	**C_8 1.3/nm²**	-5.115	1.47	1.49	0.68	1	23.5
	**C_8 2.4/nm²**	-5.587	0.50	1.49	0.83	1	25.0
**20%v-series**	**Bare**	-4.788	3.13	1.54	0.60	1	41.0
	**C_8 0.8/nm²**	-4.943	2.19	1.74	0.66	1	42.0
	**C_8 1.3/nm²**	-5.064	1.66	1.60	0.69	1	37.0
	**C_8 2.4/nm²**	-5.439	0.70	1.61	0.72	1	34.0
**30%v-series**	**Bare**	-4.763	3.32	1.93	0.61	1	47.5
	**C_8 0.8/nm²**	-4.872	2.58	1.53	0.64	1	45.8
	**C_8 1.3/nm²**	-5.115	1.48	1.61	0.69	1	47.0
		-5.064	1.66	1.50	0.69	1	47.0

The different fit contributions are shown in Figure S4.

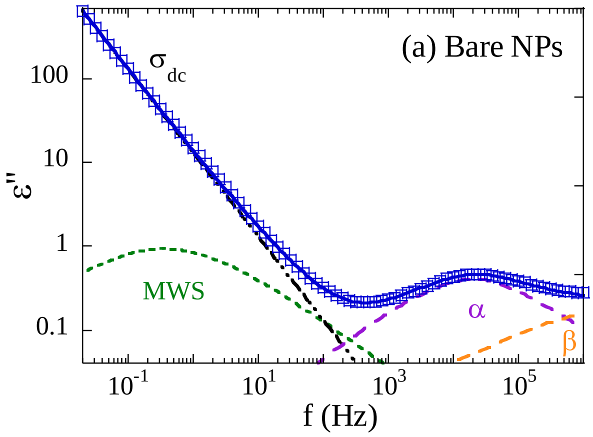

Figure S4. Frequency dependence of the imaginary part of the dielectric permittivity (squares) in 30%v-PNCs at T = 423 K for (**a**) bare NPs and (**b**) C_8 1.3 nm^{-2} grafted NPs. The solid lines represent the ILM fits as discussed in the main text, including dc-conductivity, MWS and secondary β processes (dashed and dotted lines as indicated).

IV. SAXS data of PNCs

The scattered intensities of PNCs filled with 15%v and 20%v of silica are given in Figure S5 with the corresponding apparent structure factors. Data at 30%v of silica shown in the manuscript are reproduced here for the sake of comparison.

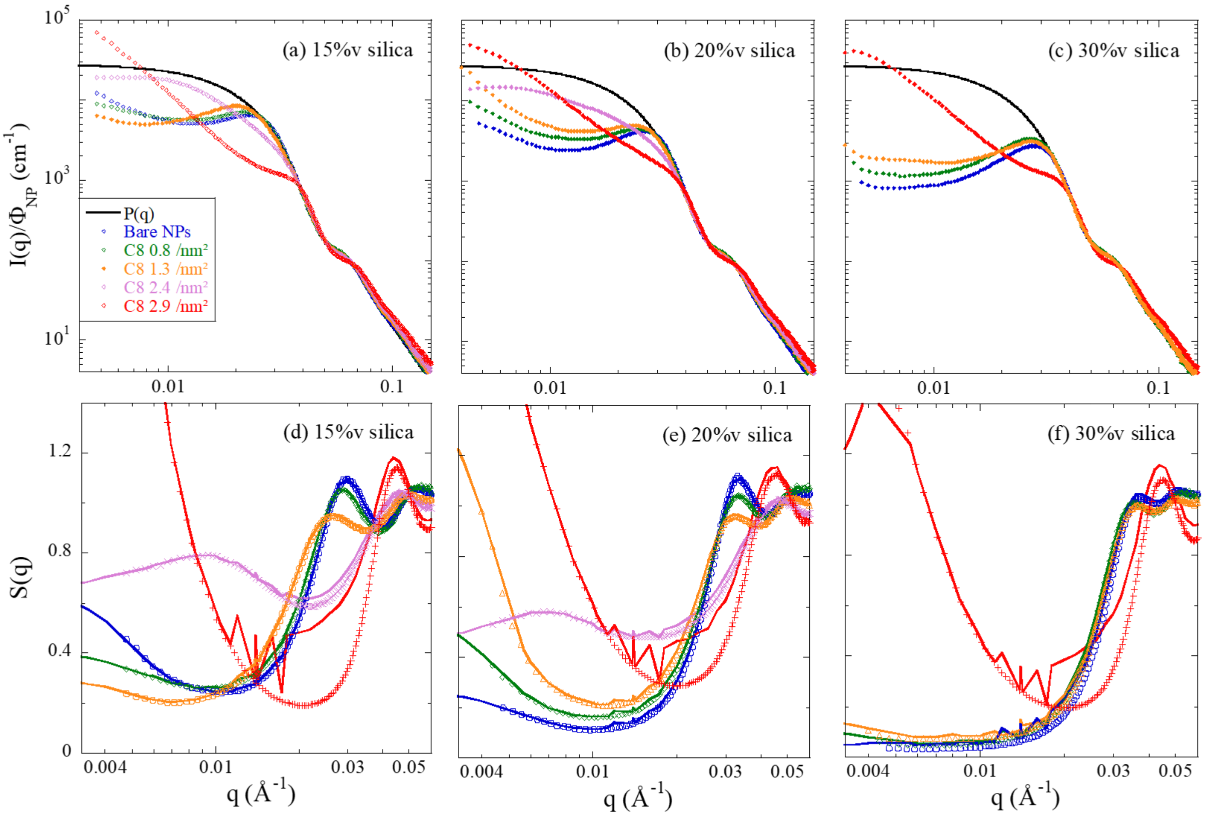

Figure S5. Top row: SAXS scattered intensities of P2VP-silica PNCs of different surface modifications for (**a**) 15%v-series, (**b**) 20%v, and (**c**) 30%v. The particle form factor is superimposed (black line). Bottom row: corresponding apparent structure factors with RMC fits (solid lines) for the series at (**d**) 15%v, (**e**) 20%v, and (**f**) 30%v.

The filler structures of two nominally identical PNCs produced at different moments are compared in Figure S6 showing a very good agreement, thus demonstrating the reproducibility of our sample preparation.

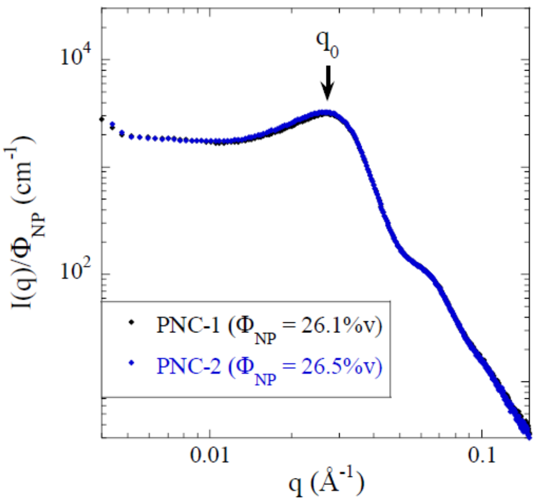

Figure S6. Comparison of the SAXS intensities of two similar P2VP-nanocomposites with C_8 surface-modified NPs (grafting density = 1.3 nm^{-2}). The arrow indicates the position of the repulsive peak.

The peak position in the scattered intensity (q_0 as exemplified in Figure S6) gives the typical center-to-center distance over which particles interact repulsively due to their hard cores: $d = 2\ \pi/q_0$. These distances are plotted in Figure S7a as a function of the grafting density and in Figure S7b as a function of the silica fraction, for all PNC samples with a well-defined peak in Figure S5.

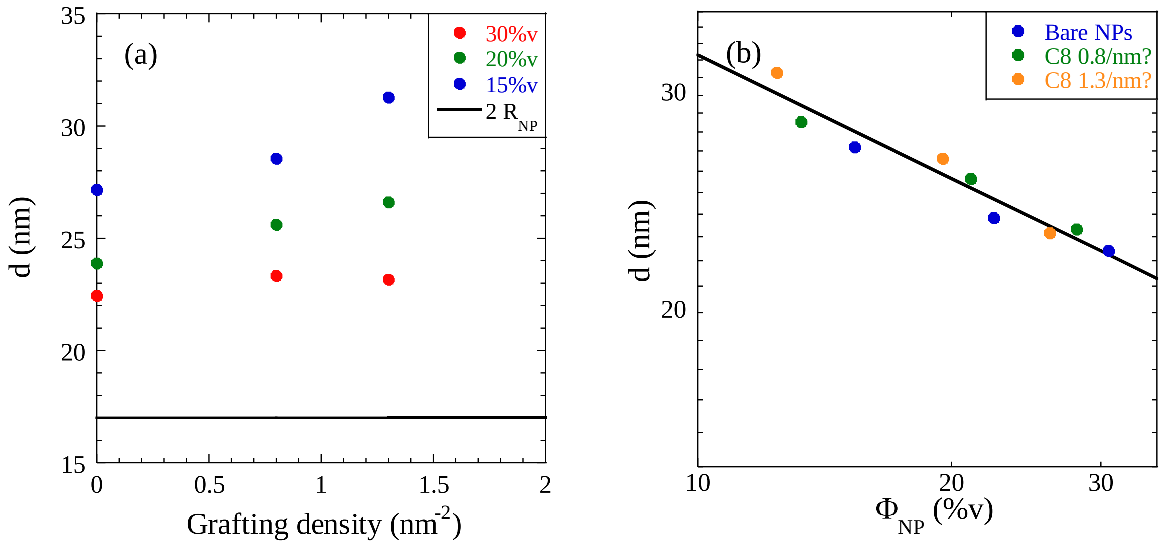

Figure S7. Center-to-center distance d associated with the peak position in Figure S4, $d = 2\pi/q_0$, as function of the grafting density (**a**) and the silica volume fraction (**b**). Log-log scale in (**b**). The solid line is a fit to a power law with exponent equal to -1/3.

The peak positions in Figure S7b follow a $\Phi^{-1/3}$-law for all PNC samples. This indicates that the slight variations observed upon grafting in the peak position can be explained by the variation in volume fraction between samples.

V. Uncertainty of IPS

The uncertainty of the interparticle spacing distributions relates to the position of the sample intensity with respect to the particle form factor in Figure S5. The latter can vary by ± 2%. To get an estimation of the IPS error bar, we have thus shifted the scattered intensity of a given sample by ± 2% and repeated the same Monte Carlo analysis for the shifted S(q). They are shown in Figure S8a for the 15%v-PNC at 1.3 nm^{-2} and the resulting normalized IPS distributions are given in Figure S8b.

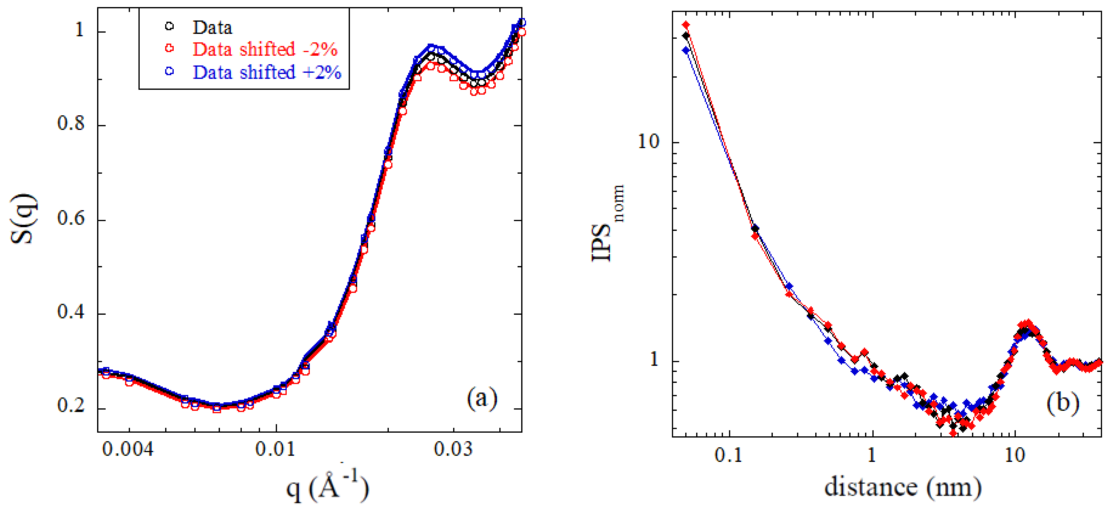

Figure S8. (**a**) Structure factor of the 15%v-PNC at 1.3 nm^{-2} considering a shifted I(q) by ± 2% (red and blue data). Solid lines are the corresponding RMC fits. (**b**) IPS vs. surface-to-surface distance, normed to the same quantity in a hard-sphere gas of same parameters, using the data in (**a**).

VI. Pair-correlation functions

The pair-correlation functions have been calculated and averaged from the positions of the particle centers in the simulation box, for the PNC series filled with 15%v of silica and different grafting densities of C_8-silane in Figure S9. As observed in [12] with C_{18}-silane, the effect of grafting is very strong on the pair-correlation function at low volume fraction of silica. Figure S9 shows that bare particles have low contact values presumably due to the presence of chains at the interface. The number of contacts strongly increases upon C_8-grafting as discussed in the manuscript.

Note that the contact values of all samples in Figure S9 are significantly lower than for the corresponding IPS in Figure 5. There is a technical reason for this: the pair-correlation function (between centers of mass) is sensitive to polydispersity, thus distributing the contact values between many radii ($R_i + R_j$). Here, we use IPS which focuses *all* contacts to a single surface-to-surface distance, d = 0.

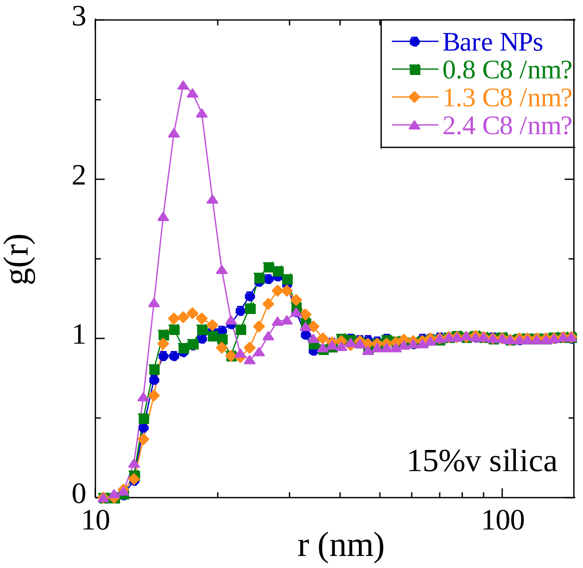

Figure S9. Pair-correlation function for the 15%v-PNCs with different grafting density of C_8-silane as indicated in the legend.

VII. Interfacial layer volume fraction curves

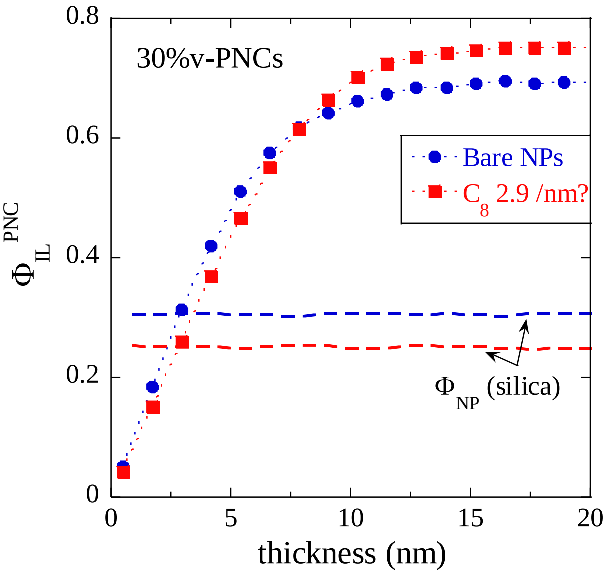

Figure S10. Volume fraction of interfacial layer Φ_{IL}^{PNC} as a function of interfacial layer thickness with respect to the entire sample ($\Phi_{IL}^{PNC} + \Phi_{NP} + \Phi_{bulk} = 1$), for 30%v-PNCs with different grafting density. Circles are bare NPs (Φ_{NP} = 30.7%v) and squares are high silane grafting (C_8 2.9 nm^{-2}, Φ_{NP} = 25.2%v). The dashed lines represent the silica volume fraction of each sample as determined by the same algorithm.

REFERENCES

12. Genix, A.-C.; Bocharova, V.; Carroll, B.; Dieudonné-George, P.; Chauveau, E.; Sokolov, A.P.; Oberdisse, J. How Tuning Interfaces Impacts Dynamics and Structure of Polymer Nanocomposites Simultaneously. *ACS Applied Materials & Interfaces* **2023**, *15*, 7496–7510, doi:10.1021/acsami.2c18083.

17. Genix, A.-C.; Bocharova, V.; Kisliuk, A.; Carroll, B.; Zhao, S.; Oberdisse, J.; Sokolov, A.P. Enhancing the Mechanical Properties of Glassy Nanocomposites by Tuning Polymer Molecular

Weight. *ACS Applied Materials & Interfaces* **2018**, *10*, 33601-33610, doi:10.1021/acsami.8b13109.
19. Kremer, F.; Schönhals, A. *Broadband Dielectric Spectroscopy*; Springer-Verlag: Heidelberg, 2003.
